# The microsurgical advantage of huge cystic craniopharyngioma following long-term cystic aspirations via Ommaya reservoir: A case report

**DOI:** 10.1097/MD.0000000000041693

**Published:** 2025-02-28

**Authors:** Sue-Jee Park, Dircia Canisia Marcelina Correia, Yeong Jin Kim, Seul-Kee Kim, Kyung-Hwa Lee, Kang-Hee Ahn, Tae-Young Jung

**Affiliations:** a Department of Neurosurgery, Chonnam National University Medical School and Hwasun Hospital, Hwasun, Republic of Korea; b Department of Medical Science, Graduate School of Biomedical Sciences, Chonnam National University Medical School, Hwasun, Republic of Korea; c Department of Radiology, Chonnam National University Medical School and Hwasun Hospital, Hwasun, Republic of Korea; d Department of Pathology, Chonnam National University Medical School and Hwasun Hospital, Hwasun, Republic of Korea.

**Keywords:** adamantinous, aspiration, craniopharyngioma, cyst, Ommaya reservoir

## Abstract

**Rationale:**

Craniopharyngiomas are histologically benign tumors with a relatively high recurrence rate. Surgical removal is challenging due to proximity to critical brain structures. This report introduces a staged operation strategy for a huge cystic craniopharyngioma.

**Patient concerns:**

An 8-year-old girl presented with diplopia and decreased visual acuity.

**Diagnoses:**

Brain magnetic resonance imaging revealed a large, 10-cm-sized cystic mass encasing both the anterior and middle cerebral arteries and the optic nerve.

**Interventions:**

An Ommaya reservoir was placed for periodic cyst aspirations over a period of 15 months. Subsequent magnetic resonance imaging indicated a reduction in cyst size, with an increasingly distinct tumor–pial interface evident on T2-weighted images. The tumor, initially entwined with neurovascular structures, gradually became delineated from these critical components. She underwent a secondary surgical intervention utilizing a bifrontal interhemispheric approach. Intraoperatively, the tumor was meticulously dissected and totally excised without compromising surrounding critical structures, while the tumor’s origin, the pituitary stalk, underwent partial resection.

**Outcomes:**

There was no worsening of vision after surgery. She was on minirin medication, and there was no recurrence during the 1-year follow-up.

**Lessons:**

Microsurgery of huge cystic craniopharyngioma following long-term cyst aspirations via Ommaya reservoir could present an efficacious strategy to diminish complication risks in pediatric patients.

## 1. Introduction

Craniopharyngiomas, accounting for approximately 2% to 5% of all pediatric intracranial tumors, originate from the embryological remnants of Rathke’s pouch.^[1]^ These tumors are predominantly found in the sellar, suprasellar, and parasellar regions.^[1,2]^ Despite their benign histological appearance, craniopharyngiomas are regarded as clinically aggressive due to their high rates of recurrence and progression.^[1,3]^ Notably, in pediatric patients, cystic adamantinomatous craniopharyngiomas are more common and present greater surgical challenges and higher recurrence rates than the papillary type of solid craniopharyngiomas predominantly seen in adults.

The surgical excision of these tumors is particularly demanding due to their proximity to vital neurovascular structures and critical neural tissue, including the optic nerve, cerebral arteries, hypothalamus, and the pituitary stalk.^[4,5]^ Consequently, the primary goal in surgical management is to achieve maximal tumor removal while preserving the patient’s long-term functional outcomes.^[6]^ Alternatively, treatments such as the placement of the Ommaya reservoir and cyst aspiration or subtotal resection (STR), followed by adjuvant radiation therapy (RT), have been suggested.^[7,8]^ However, these alternative treatments have their limitations.

This report delineates the microsurgery of huge cystic craniopharyngiomas following long-term cyst aspirations via an Ommaya reservoir. Periodic cyst aspirations of huge cystic craniopharyngioma have been reducing tumor volume. This reduction in tumor volume facilitates the separation of critical structures encased by the tumor, resulting in an increasingly distinct tumor–pial interface both radiologically and intraoperatively. This approach could present an effective strategy to reduce postoperative complication risks.

## 2. Case presentation

**Figure 1.**
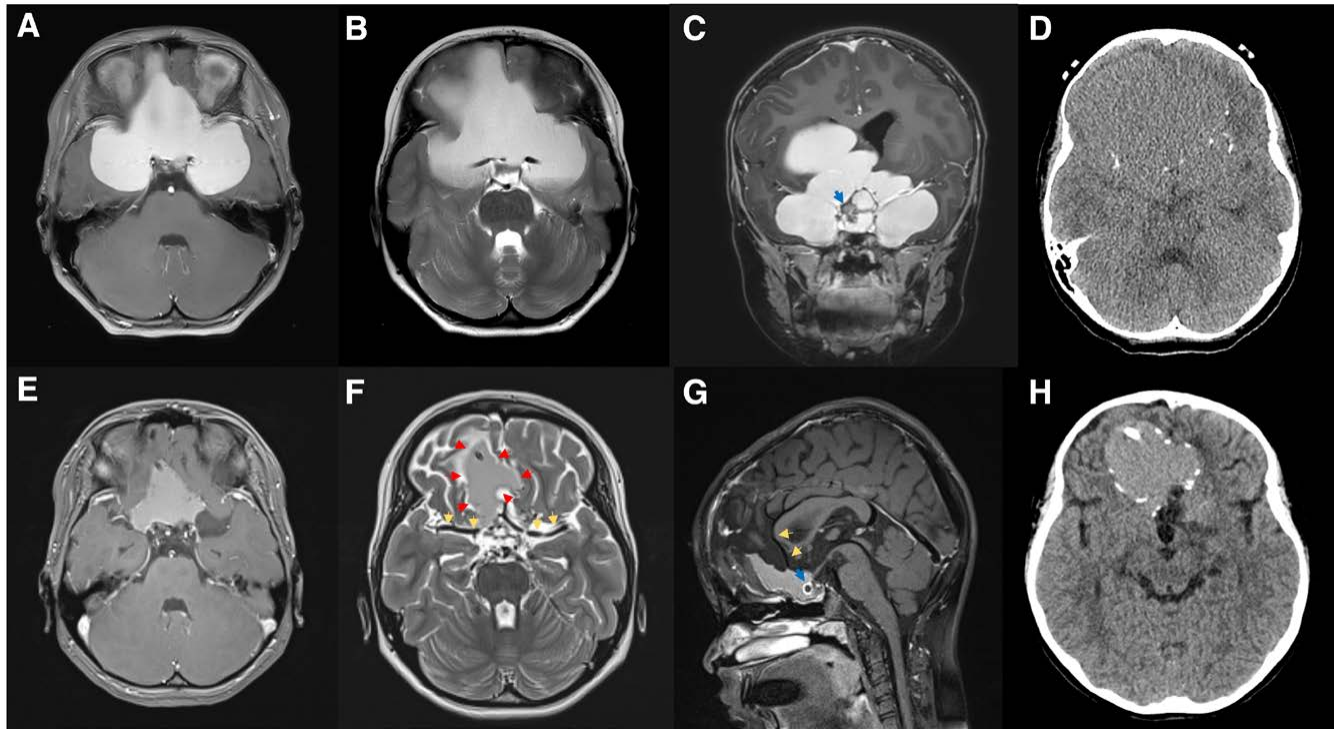
The radiologic findings of cystic craniopharyngioma following long-term cystic aspirations via the Ommaya reservoir. Preoperative brain magnetic resonance imaging (MRI) revealed a 10 cm-sized cystic mass involving both the anterior and middle cranial fossae. The cyst shows hyperintensity signals on (A) T1-weighted MRI and (B) T2-weighted MRI. (C) On contrast-enhanced T1-weighted MRI, the cyst, which was not enhanced by gadolinium, encased both optic nerves, the anterior cerebral artery (ACA), and the middle cerebral artery (MCA), and was accompanied by a solid mass (blue arrow) in the suprasellar area, suggesting a craniopharyngioma rather than a neurenteric cyst. (D) Brain computed tomography (CT) shows fine calcification along the cyst wall. Following long-term cystic aspirations through the Ommaya reservoir, the cyst shrank to 4.3 cm in size on (E) T1-weighted MRI. The tumor–pial plane (red arrows) became more distinct, and both MCAs were separated from the cyst (yellow arrows) on (F) T2-weighted MRI. The cyst was also separated from both ACAs (yellow arrows) and accompanied by a solid mass (blue arrow) on (G) contrast-enhanced T1-weighted MRI. (H) Brain CT, performed after cyst aspiration, shows more fine calcification along the cyst wall compared to the previous scan. ACA = anterior cerebral artery, CT = computed tomography, MCA = middle cerebral artery, MRI = magnetic resonance imaging.

**Figure 2.**
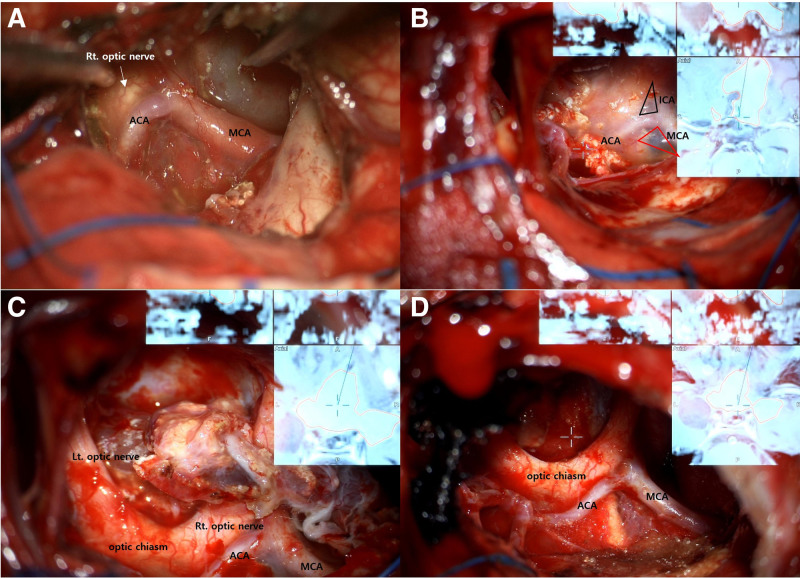
The microsurgical finding of cystic craniopharyngioma following long-term cystic aspirations via the Ommaya reservoir. (A) The initial surgical finding was that the cyst was filled with dark yellow fluid containing crystals, and the cyst wall was found to be strongly adhered to the surrounding parenchyma, especially to the optic nerve, ACA, and MCA. Following long-term cystic aspirations through the Ommaya reservoir, intraoperative findings from the second surgery showed that (B) an arachnoid membrane was observed in the space of the opticocarotid (black triangle) and supracarotid (red triangle) triangles. The adhesions between the cystic wall and critical structures were not as severe, consistent with the delineation between the tumor and pial plane observed on imaging. In particular, (C) a calcified solid mass was found to be firmly adherent around the right optic nerve. (D) The cyst wall and calcified mass were totally removed without causing damage to the surrounding neurovascular structures. ACA = anterior cerebral artery, MCA = middle cerebral artery.

**Figure 3.**
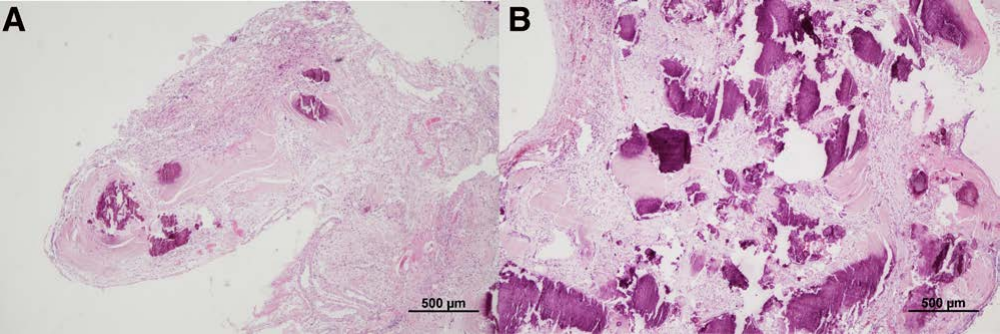
Pathology of cystic craniopharyngioma. Histopathological findings revealed anucleated squamous cells (ghost cells) with eosinophilic cytoplasm, termed wet keratin (arrows), and calcifications (arrowheads) (A) in the sample obtained from the first surgery, along with an increased density of calcifications (B) in the sample from the second surgery (hematoxylin and eosin staining, original magnification ×40).

An 8-year-old female patient presented with diplopia and reduced visual acuity, recorded at 0.2, corrected to 0.5 in the right eye, and light perception in the left eye, alongside papilledema. A brain magnetic resonance imaging (MRI) identified a 10-cm cystic mass exhibiting hyperintensity in T1 and T2-weighted images (Fig. 1A, B). The mass was not enhanced with gadolinium and encased both optic nerves, anterior cerebral artery (ACA), and middle cerebral artery (MCA), accompanied by a calcified solid mass in the suprasellar area (Fig. 1C). Brain computed tomography (CT) showed tiny calcifications along the cyst wall (Fig. 1D). During pre-operative hospitalization, she experienced a generalized tonic-clonic seizure and a decline in mental status. An emergency operation was conducted using a bifrontal interhemispheric approach with neuronavigation. Intraoperatively, the cyst was found to contain dark yellow fluid with cholesterol crystals. The cystic fluid was aspirated, revealing a thin cyst wall that was tenaciously adherent to the surrounding parenchyma and neurovascular structures, complicating the dissection (Fig. 2A). The procedure was completed by positioning the catheter within the cyst and implanting an Ommaya reservoir without forcibly detaching the cyst wall. Histopathological analysis revealed wet keratin containing ghost-like squamous cells and calcifications, leading to a diagnosis of adamantinomatous craniopharyngioma, central nervous system World Health Organization grade 1 (Fig. 3A).

After surgery, her mentality recovered, and her diplopia and visual acuity gradually improved. The patient’s visual acuity was stable at 1.0 in the right eye and hand movement in the left eye, without the requirement for pituitary hormone replacement. The fundoscopy revealed pale optic nerve heads bilaterally. Following the insertion of an Ommaya reservoir, periodic aspirations of dark yellowish cystic fluid were carried out, culminating in a total of 7 aspirations with a median aspiration volume of 12cc (range, 4–30) over a period of fifteen months leading up to a secondary surgical procedure. Follow-up brain MRI prior to the second surgery demonstrated a reduction in cyst size with the enhanced delineation between the tumor and the pial plane on T2-weighted images (Fig. 1E–G). The cyst initially enmeshed with ACA and MCA, was observed to be distinctly separated from these vascular structures. Subsequent CT revealed an escalation in calcification along the cyst wall relative to prior scans (Fig. 1H).

**Figure 4.**
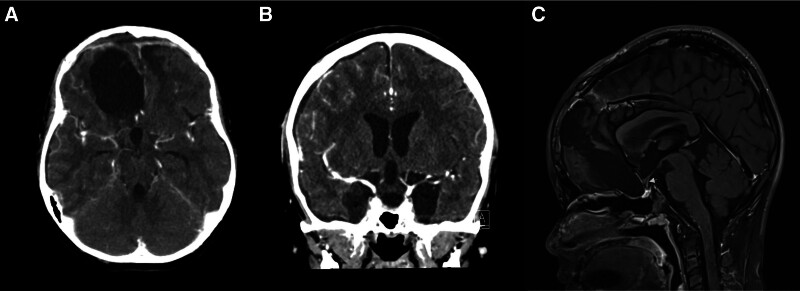
The postoperative radiologic findings and follow-up. Postoperative brain contrast-enhanced CT shows nearly complete removal of the tumor, with preservation of the surrounding parenchyma and neurovascular structures, as seen in the (A) axial and (B) coronal views. During 1-year of follow-up, there was no recurrence with the preservation of the pituitary stalk (yellow arrow) in the (C) sagittal view of MRI. CT = computed tomography, MRI = magnetic resonance imaging.

A secondary operation was performed using the bifrontal interhemispheric approach as before. Intraoperatively compared to the previous surgery, adhesions between the cystic wall and critical structures were not as severe, consistent with the delineation between the tumor and pial plane observed on imaging (Fig. 2B). The cyst wall was carefully separated using a micro bayonet. Certain areas of the thickened cyst capsule and calcified mass were firmly adherent to the right optic nerve and branches of MCA. These adherent portions were precisely dissected and removed with micro scissors (Fig. 2C). The tumor was found to originate from the pituitary stalk; through partial resection of the stalk, it was possible to excise the tumor completely without causing damage to the surrounding structures (Fig. 2D). Postoperative brain CT imaging revealed no residual tumor, and the pituitary stalk was identifiable (Fig. 4A, B). Histopathological analysis showed a diagnosis of adamantinomatous craniopharyngioma, indicating increased calcification density (Fig. 3B). After the second operation, the patient’s visual acuity was maintained, with a visual acuity of 1.0 on the right and hand motion on the left, and her consciousness was intact. She was administered minirin medication for pituitary hormone replacement while other pituitary hormones remained stable. There was no worsening of vision after surgery. There was no recurrence during the 1-year follow-up (Fig. 4C).

## 3. Discussion

It is well known that craniopharyngioma in children tends to have more cystic components compared to those in adults.^[4]^ Craniopharyngioma causes symptoms due to its mass effect, mainly resulting in bitemporal hemianopsia, hormone deficiencies, or hydrocephalus. Although these craniopharyngiomas are classified as benign tumors, central nervous system World Health Organization grade 1, these are associated with a high incidence of recurrence and progression.^[2,9]^ According to some studies, the progression-free-survival rates for patients treated with gross total resection (GTR) were 85% and 75% at 2 and 5 years, as opposed to 20% for STR alone at 5 and 10 years.^[2]^ Therefore, it is recommended to perform GTR, but due to adhesion with adjacent nervous tissue and neurovascular structures, GTR is sometimes not feasible.^[4,5]^ Hence, in light of these challenges, alternative treatments for craniopharyngioma have been suggested.

Alternatively, treatments such as the placement of the Ommaya reservoir and cyst aspiration or STR, followed by adjuvant RT, have been suggested.^[7,8]^ However, these alternative treatments have their limitations. Periodic cyst aspiration through the Ommaya reservoir ultimately proved ineffective in preventing tumor progression.^[4]^ In 80% of cases, tumor progression persisted, and preoperative neurological deficits did not improve.^[4]^ Moreover, RT after STR in children should be approached with extreme caution. This is primarily due to the potential risks of developing neurocognitive impairment, radiation-induced vasculopathy, and secondary malignancies.^[2,10]^ This might result in both short-and long-term morbidities. Therefore, it is crucial to conduct as maximal safe resection of craniopharyngioma as possible without damaging surrounding neurovascular structures.^[6]^

In this case, the periodic cyst aspiration subsequent to the Ommaya reservoir placement has been instrumental in diminishing the tumor volume, which strategy had revealed notable intraoperative advantages. The volume reduction facilitated the separation of critical structures previously encased by the tumor. Such a procedural strategy diminishes the risk of damage to these vital structures during the surgical intervention and streamlines the overall surgical process. Additionally, the reduced tumor volume significantly curtails the necessity for extensive brain retraction, thereby mitigating the possibility of inadvertent injury to the adjacent parenchyma and neurovascular structures. A notable observation in our case, with the cyst shrinkage, was the enhanced delineation of the tumor–pial interface in the T2-weighted MRI (Fig. 1F). As corroborated by the T2-weighted MRI (Fig. 1B, F), in the initial surgery, the delicate cystic wall, presenting with extensive adhesions, spanned the optic nerve, ACA, and MCA (Fig. 2A). However, during the second surgery, an arachnoid membrane was observed in the space of the opticocarotid and supracarotid triangle, allowing meticulous dissection of a portion of the cyst wall (Fig. 2B). When the tumor exhibits minimal adhesion to the adjacent structures and the cystic capsule is amenable to gentle peeling, the possibility of minimizing the risk of causing collateral tissue damage escalates. After periodic cyst aspirations, the reduction in tumor volume facilitates the separation of critical structures encased by the tumor, resulting in an increasingly distinct tumor–pial interface both radiologically and intraoperatively. Consequently, the strategy of cyst size reduction via Ommaya reservoir aspiration, coupled with a staged surgical approach, emerges as a viable alternative surgical strategy aiming at minimizing the risk of mortality and morbidity.

Despite the potential insights offered by this case report, it is limited by the brief follow-up duration and being a single case study. There is a pressing need to explore a broader spectrum of cases involving such rare cysts and to explore additional therapeutic strategies. These efforts are necessary to help define an evidence-based set of guidelines for the management of pediatric patients with cystic craniopharyngioma.

## 4. Conclusion

Cystic craniopharyngioma is a benign tumor that mainly occurs in children. Due to its high recurrence and progression rates, it is crucial to perform maximal safe resection of the tumor to minimize the risk of recurrence. We suggest that the microsurgery of huge cystic craniopharyngioma following long-term cyst aspirations via the Ommaya reservoir could present an efficacious strategy to diminish complication risks in pediatric patients.

## Author contributions

**Conceptualization:** Tae-Young Jung.

**Data curation:** Sue-Jee Park, Kyung-Hwa Lee, Tae-Young Jung.

**Writing – original draft:** Sue-Jee Park, Seul-Kee Kim.

**Writing – review & editing:** Dircia Canisia Marcelina Correia, Yeong Jin Kim, Kyung-Hwa Lee, Kang-Hee Ahn, Tae-Young Jung.

## References

[1] Aldave G Okcu MF Chintagumpala M. Comparison of neurocognitive and quality-of-life outcomes in pediatric craniopharyngioma patients treated with partial resection and radiotherapy versus gross-total resection only. J Neurosurg Pediatr. 2023;31:453–62. 36806176 10.3171/2022.12.PEDS22367 PMC10193466

[2] Fouda MA Karsten M Staffa SJ Scott RM Marcus KJ Baird LC. Management strategies for recurrent pediatric craniopharyngioma: new recommendations. J Neurosurg Pediatr. 2021;27:548–55. 33668031 10.3171/2020.9.PEDS20606

[3] Fouda MA Riordan CP Zurakowski D Goumnerova LC. Analysis of 2141 pediatric craniopharyngioma admissions in the USA utilizing the Kids’ Inpatient Database (KID): predictors of discharge disposition. Childs Nerv Syst. 2020;36:3007–12. 32363544 10.1007/s00381-020-04640-4

[4] Greuter L Richards OL Malik N. Supraorbital minicraniotomy for open Ommaya reservoir placement in pediatric craniopharyngiomas: a case series and technical report. J Neurosurg Pediatr. 2023;32:421–7. 37410604 10.3171/2023.5.PEDS2390

[5] Watanabe T Uehara H Takeishi G. Proposed system for selection of surgical approaches for craniopharyngiomas based on the optic recess displacement pattern. World Neurosurg. 2023;170:e817–26. 36481441 10.1016/j.wneu.2022.11.138

[6] Prieto R Pascual JM Hofecker V. Craniopharyngioma adherence: a reappraisal of the evidence. Neurosurg Rev. 2020;43:453–72. 30043262 10.1007/s10143-018-1010-9

[7] Frio F Solari D Cavallo LM Cappabianca P Raverot G Jouanneau E. Ommaya reservoir system for the treatment of cystic craniopharyngiomas: surgical results in a series of 11 adult patients and review of the literature. World Neurosurg. 2019;132:e869–77. 31400528 10.1016/j.wneu.2019.07.217

[8] Lohkamp LN Kulkarni AV Drake JM. Preservation of endocrine function after Ommaya reservoir insertion in children with cystic craniopharyngioma. J Neurooncol. 2022;159:597–607. 35925530 10.1007/s11060-022-04099-0

[9] Maia R Miranda A Geraldo AF. Neuroimaging of pediatric tumors of the sellar region-A review in light of the 2021 WHO classification of tumors of the central nervous system. Front Pediatr. 2023;11:1162654. 37416813 10.3389/fped.2023.1162654 PMC10320298

[10] Cohen M Bartels U Branson H Kulkarni AV Hamilton J. Trends in treatment and outcomes of pediatric craniopharyngioma, 1975-2011. Neuro Oncol. 2013;15:767–74. 23486689 10.1093/neuonc/not026 PMC3661103

